# Benefits of cetalkonium chloride cationic oil-in-water nanoemulsions for topical ophthalmic drug delivery

**DOI:** 10.1111/jphp.12075

**Published:** 2013-05-26

**Authors:** Philippe Daull, Frédéric Lallemand, Jean-Sébastien Garrigue

**Affiliations:** Novagali PharmaEvry, France

**Keywords:** cationic, cetalkonium chloride, drug delivery, oil-in-water emulsion, ocular surface

## Abstract

**Objectives:**

Topical ocular administration is the most convenient route of administration of drugs for the treatment of eye diseases. However, the bioavailability of drugs following eye instillations of eye drops is very low. Over the past 20 years, extensive efforts have been put into research to improve drug bioavailability without compromising treatment compliance and patients' quality of life.

**Key findings:**

One of the most efficient ways to improve drug bioavailability is to increase the precorneal residence time of the eye drop formulations. As a result, new eye drops, with bioadhesive properties, have been developed based on the cationic oil-in-water (o/w) nanoemulsion technology. These low viscosity eye drop nanoemulsions have improved precorneal residence time through the electrostatic interactions between the positively charged oil nanodroplets and the negatively charged ocular surface epithelium.

**Summary:**

This review is the first to present the benefits of this new strategy used to improve ocular drug bioavailability. The roles of the cationic agent in the stabilization of a safe cationic o/w nanoemulsion have been discussed, as well as the unexpected benefits of the cationic o/w nanoemulsion for the protection and restoration of a healthy tear film and corneal epithelium.

## Introduction

Aqueous eye-drop solutions are still the most common formulations for topical ocular drug delivery, since they are the simplest, easiest and cheapest ocular dosage forms to produce. The main drawbacks of these conventional ocular dosage forms are that they are rapidly eliminated from the ocular surface following instillation, resulting in a low ocular bioavailability (less than 1%) of the drugs, and are limited to water soluble compounds.^1^ Variants with viscosifying agents, penetration enhancers or spreading surfactants have not fundamentally changed the paradigm. Suspensions, gels and negatively charged oil-in-water (o/w) nanoemulsions were developed to improve ocular bioavailability of lipophilic or poorly water soluble drugs.^2^–^4^ Among them, o/w nanoemulsions were demonstrated to be effective ocular drug delivery vehicles.^5^,^6^

Anionic o/w nanoemulsions with ciclosporin (cyclosporine A; Restasis, Allergan) were developed to increase tear production in patients whose tear production was presumed to be suppressed due to ocular inflammation (i.e. for patients with dry eye disease), or with difluprednate (Durezol, Alcon) for the treatment of inflammation and pain associated with eye surgery.^7^ Oil-in-water nanoemulsions were demonstrated to be excellent vehicles for lipophilic drugs, such as ciclosporin or prostaglandin analogues like latanoprost or tafluprost, but also for delivering water unstable drugs.^8^,^9^

Cationic o/w nanoemulsions extended one step further the benefits of the o/w nanoemulsions for drug delivery by improving their residence time over that observed with the anionic o/w nanoemulsions. These cationic o/w nanoemulsions take advantage of the negatively charged ocular surface to increase through electrostatic interactions their precorneal residence time, and thus the ocular drug bioavailability.^10^,^11^ As a consequence, the first generation of cationic o/w nanoemulsions were developed to optimize penetration of drugs (among them ciclosporin) in ocular tissues.^9^,^11^–^14^ This first generation of cationic o/w nanoemulsions used noncompendial cationic surfactants and were not devoid of ocular toxicity side effects.^15^–^17^ Hence, the challenges for the development of cationic o/w nanoemulsions are in the choice of the most appropriate cationic agent used to bring the positive charge to the oil nanodroplets, and in the improvement of the ocular tolerance of these positively charged nanoemulsions.

This review is the first to compile the information present in the literature that describes this new strategy used to improve ocular drug delivery: the use of cationic o/w nanoemulsion vehicles in ocular drug delivery. The main steps involved in the pharmaceutical development will be discussed, particularly the ones that led to the choice of cetalkonium chloride (CKC) as a cationic agent compatible with the ocular surface.

## Definition of a cationic oil-in-water nanoemulsion

**Figure 1:**
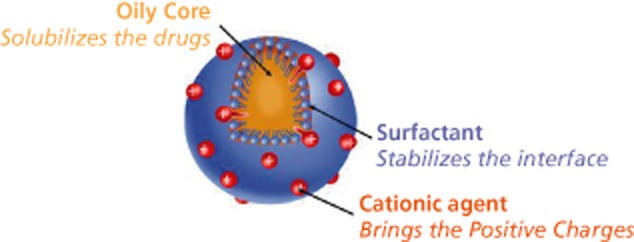
Schematic representation of one of the oil nanodroplets present in the cationic oil-in-water nanoemulsion.

By definition a cationic o/w nanoemulsion is a biphasic formulation that comprises positively charged oil nanodroplets (the oil phase) dispersed in water (the continuous phase).^18^ Table 1 summarizes the physicochemical properties of a cationic o/w nanoemulsion. The positive charge of the oil nanodroplets is brought by a cationic surfactant that localizes itself at the oil interface. Ideally, this cationic agent should be sufficiently lipophilic to be almost exclusively entrapped in the oil with only very low amounts of the cationic agent present in the aqueous phase of the formulation. In addition to the biological effects of the cationic o/w nanoemulsion (discussed below), the positive charge of the oil nanodroplets helps improve the long-term stability of the nanoemulsion by generating a repulsive electrostatic force (measured by the zeta potential) between the positively charged oil nanodroplets, thus preventing their merging and avoiding the coalescence process of the nanoemulsion during shelf life.^18^–^20^ Figure 1 presents the sketch of one of the oil nanodroplets present in the cationic o/w nanoemulsion.

**Table 1:** Summary of the physicochemical characteristics of a cationic oil-in-water nanoemulsion

Parameter	Description
Aspect	White opaque to slightly translucent
pH	5.0–7.0
Osmolality (mOsmol/kg)	270
Droplet size (nm)^a^	< 200
Zeta potential (ζ, mV)^a^	Positive (+40)
Sterility	Sterile

a Droplet size was determined by dynamic light scattering (HPPS, Malvern Instruments), and zeta potential by electrophoretic mobility measurement (Zetasizer 2000, Malvern Instruments).

While the oil phase of the nanoemulsion is generally made of inert and stable oils, such as medium chain triglycerides or mineral oil (i.e. non-vegetable liquid paraffin), the choice of the right cationic agent needed to produce a safe and well tolerated cationic o/w nanoemulsion necessitated a thorough examination of the cationic agents at hand.^21^

## Choice of the cationic agent

The positive charge of a cationic o/w nanoemulsion is estimated by measuring the zeta potential. The zeta potential (ζ) is the electrical potential difference (ΔV) between the dispersion medium (i.e. water) and the stationary layer of fluid attached to the dispersed oil nanodroplets.^22^,^23^ The zeta potential is a measure of the magnitude of the electrostatic or charge repulsion between the oil nanodroplets, and is one of the fundamental parameters known to affect the stability of dispersed systems (i.e. o/w nanoemulsion); thus the higher the zeta potential, the better (ζ ≥ +40 mV).^22^ As a consequence, to obtain a high zeta potential for the cationic o/w nanoemulsion, all, to almost all the cationic agent has to be entrapped in the oil nanodroplet, with the positive charge located at the oil–water interface, and no to very low amounts of freely soluble molecules of the cationic agent present in the aqueous phase (i.e. the dispersion medium), where they can contribute to ‘shield’ and reduce the zeta potential of the nanoemulsion. Thus, the cationic agent needs to be lipophilic, i.e. amphiphilic; and the higher the lipophilicity the better.

A large number of cationic agents were described in the literature that could have been potential cationic agents for the cationic o/w nanoemulsion, such as: stearylamine, oleylamine, poly(ethylenimine), poly(l-lysine), 1,2-di-(9Z-octadecenoyl)-sn-glycero-3-phosphoethanolamine (DOPE), 1,2-di-(9Z-octadecenoyl)-3-trimethylammonium-propane (DOTAP), and alkyl benzyldimethylammonium compounds of various alkyl chain lengths (IUPAC name: benzyl(dimethyl)azanium; but better known under their common name: benzalkonium chloride (BAK) derivatives). However, they were all hampered either by toxicity, stability or regulatory issues which avoided or limited their use in ophthalmologic products.^16^,^23^,^24^ Consequently, the search for the appropriate cationic agent was limited to the ones already registered, used in ophthalmic products or compliant with either the United States (US) or European (EU) pharmacopoeias.^11^

The most common cationic agents found in ophthalmic products belong to the family of quaternary ammoniums, such as BAK or polycationic polymers also known as polyquaternium (e.g. Polyquaternium-1 (PQ-1) found in Polyquad). However, these quaternary ammonium derivatives are used as preservative agents in conventional ocular drug products for their bactericidal and microbicidal properties (through a detergent action (see Furrer *et al*.^25^)) at relatively low concentrations in aqueous solution: 0.001% PQ-1 in Travatan (Alcon) or 0.02% BAK in Xalatan (Pfizer). Over the past twenty years, a very large collection of evidence has been published demonstrating the deleterious effects of quaternary ammonium preserved eye drop solutions for the ocular surface, especially for BAK-preserved solutions.^26^–^33^ The actual trend for conventional ocular drug products– i.e. for eye drop aqueous solutions– is to reduce the concentration, or even remove completely quaternary ammoniums, and especially BAK, from their compositions.^34^ As a consequence many soft-preserved and preservative-free ophthalmic drug products have reached the market in the last few years.^35^ Hence, can quaternary ammonium chlorides, and among them BAK derivatives still make good cationic agents for cationic o/w nanoemulsions?

## BAK derivatives as cationic agent for cationic oil-in-water nanoemulsion

As mentioned previously, the ideal cationic agent should be lipophilic enough to localize itself exclusively within the oil nanodroplets with no freely soluble cationic agent molecule within the aqueous phase for better zeta potential and shelf life stability, and improved safety profile.^22^,^36^

**Figure 2:**
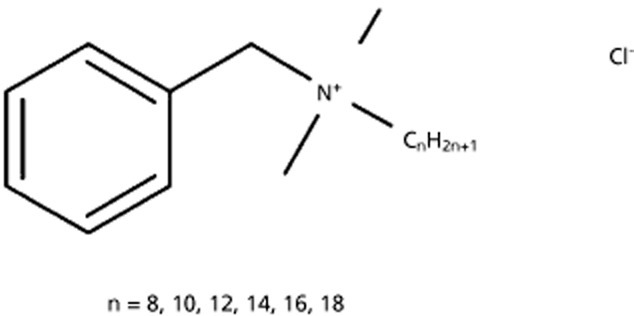
Benzalkonium chloride is a mixture of alkyl benzyldimethylammonium chloride compounds of various chain lengths.

According to the latest (2012) US and EU pharmacopoeias BAK is a mixture of alkyl benzyldimethyl quaternary ammonium chlorides of various alkyl chain lengths (Figure 2). The alkyl chains are ranging from 8 to 18 carbons, with the C12, C14 and C16 alkyl derivatives being the most common in the BAK mixture (Figure 2). Indeed, the pharmacopoeias specify that the BAK mixture content of the C12 homologue should not be less than 40%, and the content of the C14 homologue not less than 20% of the total alkyl benzyldimethylammonium chloride content. In addition the sum of the C12 and C14 alkyl derivatives has to represent at least 70% of the BAK composition. Note that even for pharmacopoeia compliant BAK mixtures, the composition and distribution of the alkyl chain derivatives can vary from one manufacturer to another.^37^ Thus, is there among these alkyl benzyldimethylammonium of various alkyl chain lengths an alkyl derivative that possesses physicochemical properties that would make it compatible with the ideal cationic agent for an o/w cationic nanoemulsion?

## Physicochemical properties of the C12, C14 and C16 BAK derivatives

**Figure 3:**
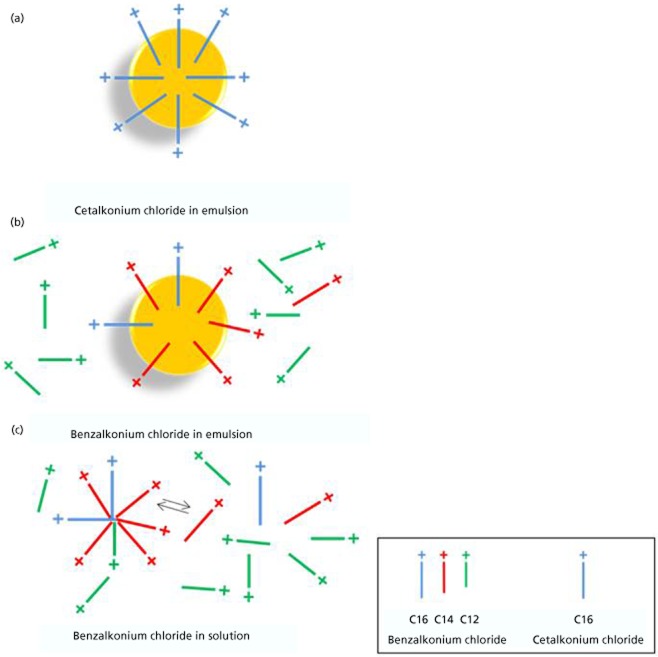
Illustration of the phase distribution for the different alkyl derivatives of benzalkonium chloride. (a) Cetalkonium chloride (blue) in emulsion; (b) benzalkonium chloride (BAK) mixture in emulsion; and (c) BAK mixture in aqueous solution.

The C12, C14, and C16 alkyl derivatives of BAK are also known as benzododecinium chloride, myristalkonium chloride, and cetalkonium chloride (CKC), respectively. Table 2 summarizes the physicochemical properties of theses alkyl derivatives. It appears that CKC is the most lipophilic and less water soluble among the three major BAK derivatives present in the BAK mixture. Thus, the C16 BAK derivative (i.e. CKC) with a calculated logP of 9.5 is a particularly attractive cationic agent for cationic o/w nanoemulsions. Figure 3 illustrates the phase distribution for the different alkyl derivatives of BAK in o/w nanoemulsions or aqueous solution. Due to their lower lipophilicity, low concentrations of the C12 and C14 BAK derivatives can be found in the aqueous phase of the cationic o/w nanoemulsion, while no C16 BAK derivatives (i.e. CKC) are present in the aqueous phase of the o/w nanoemulsion. This is confirmed by the measure of the zeta potential. For cationic o/w nanoemulsions with BAK (0.02%) as cationic agent the zeta potential is approximately +20 mV, while for a cationic o/w nanoemulsion with CKC (0.005%) as the cationic agent the zeta potential is +40 mV.^38^,^39^

**Table 2:** Physicochemical properties of C12, C14 and C16 alkyl derivatives of benzalkonium chloride

BAK derivatives	C12	C14	C16 (CKC)
Molecular weight (g/mol)	340	368	396
XlogP3 (PubMed compound)	7.4	8.4	9.5
logP (calculated)	3.44	4.45	5.46
logP (measured)^a^	−0.17 < logP <−0.07	0.24 < logP < 0.44	2.4 < logP < 2.6
Critical micellar concentration (CMC)^b^	4.5 mm	0.75 mm	0.55 mm
	1.53 g/l	0.29 g/l	0.022 g/l
	0.153%	0.029%	0.0022%
Superficial tension at CMC (mN/m)	38	38	40
Water solubility (g/l; 25°C)	1230	100	8.5

BAK, benzalkonium chloride; CKC, cetalkonium chloride.

a Maximum solubility ratios in both organic and aqueous phases.

b Measured with a Wilhelmy blades tensiometer.

However, since these cationic BAK derivatives are surfactants with detergent properties and cellular membrane toxicity, as such, the BAK mixture is very often added to eye drop aqueous solutions for their preservation.^40^ It was described by Kurup *et al*.^41^ that only the free forms of the preservative (i.e. BAK derivatives) present in the aqueous phase of an o/w nanoemulsion were available for antibacterial activity, and therefore exerted ocular surface cell membrane toxicity.^42^,^43^ Thus, it is very important to have the lowest concentration of free/micelle BAK derivative molecules in the aqueous phase to avoid or limit as much as possible the side effects induced by these free molecules. Hence, for a better ocular tolerance and safety profile of the cationic o/w nanoemulsion, the higher the lipophilicity of the cationic agent, the better. Consequently, CKC was selected as the cationic agent of choice for the development of unpreserved, well tolerated cationic o/w nanoemulsions as it is the most lipophilic BAK derivative present in the BAK mixture. The following sections will discuss the ocular tolerance and safety profile of the unpreserved CKC-containing cationic o/w nanoemulsions, and the various advantages brought by CKC and its positive charge for the improvement of drug bioavailability and the protection and healing of the ocular surface.

## Biological properties of cationic oil-in-water nanoemulsion eye drops

The rationale for developing cationic o/w nanoemulsion eye drops arose from the observation that the ocular mucosa is negatively charged. Both the corneal and conjunctival human cells harbour O-glycosylated transmembrane mucins with only 6% of their glycans not terminated by the negatively charged sialic acid.^10^ When a cationic o/w nanoemulsion eye drop is instilled onto the ocular surface, the resultant electrostatic attraction between the positively charged oil nanodroplets and the ocular surface manifests itself macroscopically by an improved spreading of the eye drop preparation onto the eye.^11^ This was evidenced by dynamic contact angle measurements, which were rapidly very low with cationic o/w nanoemulsion (below 3° within the first second) upon instillation, while it remained elevated (above 42°) with either the anionic nanoemulsion or the hyaluronate hydrogel.^17^ The electrostatic interactions help to increase the residence time of the oil nanodroplets on the ocular surface. The ocular residence time plays a major role for drug absorption, and various strategies were developed to improve drug residence time as a means to improve drug absorption, such as ophthalmic inserts, viscosity enhancers/mucoadhesives, anionic o/w nanoemulsions, and cationic o/w nanoemulsions.^4^ Classic eye drop solutions are eliminated within minutes following administration, thus greatly reducing drug ocular bioavailability, which seldom exceed 1% of the delivered dose.^44^ The beneficial role of the cationic o/w nanoemulsion on the ocular bioavailability was demonstrated with ciclosporin.^45^ Cationic and anionic nanoemulsions of 0.05% ciclosporin absorptions in the conjunctiva and cornea following a single ocular application in the rabbit eye were measured over time (up to 72 h). The pharmacokinetic data demonstrated that ciclosporin's area under the curve (AUC) with the cationic o/w nanoemulsion was approximately twice the one observed with the anionic o/w nanoemulsion (AUC: 26477 vs 14210 ng/g.h, for the cationic vs anionic nanoemulsion, respectively). The maximum ciclosporin concentration (*C_max_*) in the cornea for the cationic o/w nanoemulsion following instillation was 1371.8 ng/g, while it was only 747.8 ng/g for the anionic o/w nanoemulsion.^46^ Interestingly, a second peak of ciclosporin absorption was observed in the cornea with the 0.05% ciclosporin cationic o/w nanoemulsion two hours post instillation (and even 11 h after instillation with the 0.1% ciclosporin cationic o/w nanoemulsion), while no such peak was observed with the anionic o/w nanoemulsion.^17^,^46^ The presence of this second peak is a strong argument in favour of an extended ocular residence time with cationic o/w nanoemulsions. The extended ocular residence time of the cationic o/w nanoemulsion was suggested following the instillation of a latanoprost-loaded cationic o/w nanoemulsion.^47^

## Safety profile of the cationic oil-in-water nanoemulsions

**Figure 4:**
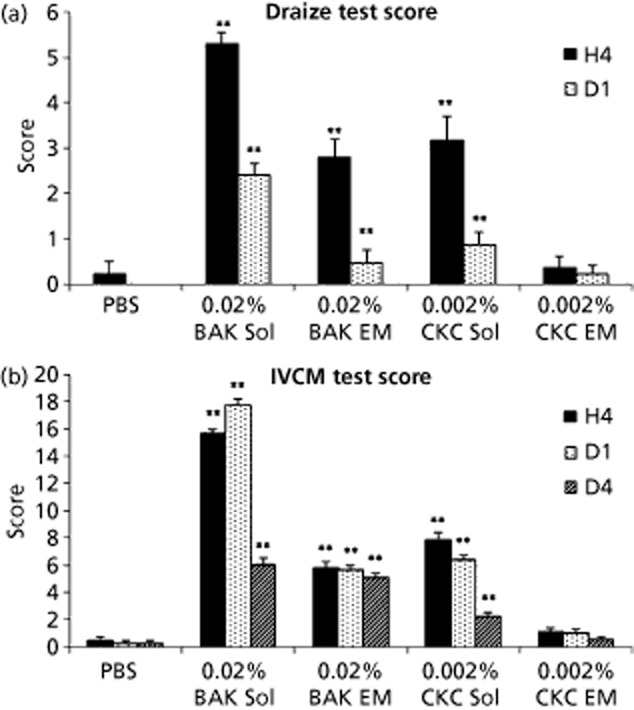
(a) Draize test score and (b) in-vivo confocal microscopy (IVCM) score of cationic oil-in-water nanoemulsion, at four hours (H4), day 1 (D1) and day 4 (D4) after the instillations. **P < 0.01 against phosphate buffered saline (PBS; nonparametric comparisons (Mann-Whitney)). BAK, benzalkonium chloride; CKC, cetalkonium chloride; Sol, solution; EM, nanoemulsion.

Adapted from Liang *et al*.^36^.

As indicated previously, the positive charge of the cationic o/w nanoemulsions is brought by CKC, a quaternary ammonium that is structurally closely related to BAK. CKC's alkyl chain contains 16 carbons. This seemingly slight difference in alkyl chain length has a major impact on the physicochemical properties of CKC (Table 2) and as a consequence on the safety profile of the CKC-containing cationic o/w nanoemulsions when compared with BAK-containing cationic o/w nanoemulsion. Liang *et al*.^36^ used an in-vivo rabbit model to demonstrate that both BAK and CKC cationic o/w nanoemulsions were much better tolerated by the rabbit ocular surface than their solution counterparts. When BAK is in solution the C12 and C14 alkyl derivatives form micelles that have the possibility to interact with the corneal and conjunctival cell membranes once applied on the ocular surface. Through their detergent properties the C12 and C14 BAK alkyl derivatives alter the epithelial cells integrity, leading to the well-known deleterious effects of BAK solutions. However, when formulated in an o/w nanoemulsion, a significant part of these C12 and C14 alkyl derivatives of BAK is entrapped in the oil (as a consequence of the lipophilicity of the C12 and C14 alkyl chains of BAK). Only a small proportion of the C12 and C14 alkyl derivatives of BAK remain in the aqueous phase of the solution, thus greatly reducing the ocular toxicity of the BAK o/w nanoemulsion (Figure 4). This is emphasized with CKC, the C16 alkyl derivative of BAK. Due to its extreme lipophilicity, when formulated in emulsion almost all of the CKC is entrapped in the oil with no free CKC molecules present in the aqueous phase of the emulsion. As a result, an even better safety profile of the cationic o/w nanoemulsion can be expected with CKC as the cationic agent rather than with BAK (Table 3)^48^–^51^. This is confirmed *in vivo* by the improved ocular tolerance of the CKC cationic nanoemulsion over the BAK cationic nanoemulsion.^36^ In a rabbit model of acute toxicity, 15 instillations of CKC-containing cationic o/w nanoemulsion had a better Draize test score and a lower in-vivo confocal microscopy (IVCM) score than the BAK-containing cationic o/w nanoemulsion (Figure 4), and were equivalent to the BAK-free saline control. The ciclosporin-containing CKC cationic o/w nanoemulsion was as well tolerated as –if not better than– the BAK-free Restasis.^52^

**Table 3:** Summary of the physical and biological properties of benzalkonium chloride solutions and cationic oil-in-water nanoemulsions

	Aqueous solutions of BAK (conventional ocular dosage forms)	Cationic oil-in-water nanoemulsions with	
		BAK (C12 +C14)	CKC (C16)
Solubility in water	Soluble	Soluble	Poorly soluble to insoluble
Solubility in oil	Not applicable (aqueous solution)	Soluble	Soluble
Zeta potential	/	∼+20 mV	∼+40 mV
Structural organization	**Micelles** (10–20 nm)	**Emulsion** (oil nanodroplet: 150–200 nm)	
Stability	Unstable (dynamic equilibrium)	Stable (++)	Stable (+++)
Localization in formulation	Water free-flowing molecules in equilibrium with micellar structures	In the oil nanodroplets and a small proportion in the aqueous phase	Bound in the oil nanodroplets
Function in formulation	**Preservative role** (resulting from the free-flowing BAK molecules in the aqueous phase^41^	**Cationic surfactant role**	
	Help solubilize lipophilic drugs	– Stabilizing the nanoemulsion – Bringing positive charge **No preservative role**^41^	
Effects	Dose-dependent **detergent effect** with destabilization of biological membranes: – Microbicidal agent – Irritancy of tissues	**No detergent effect** (most of the BAK is in the oil droplets)^41^,^43^	**No detergent effect** (CKC bound to the oil droplets)^41^,^43^
Preservative action	**Preserved** eye drop from 0.004% to 0.025% depending on formulation (0.005% in Lumigan; 0.02% in Xalatan)	**Unpreserved** cationic oil-in-water nanoemulsion eye drop	
Nonclinical results 26,36	**Toxic for the ocular surface** with ocular irritation, inflammation and apoptosis	**Not toxic for the ocular surface** No ocular irritation, no inflammation and no apoptosis As safe as saline solution	
Clinical effect 17	**Tear film instability** – Lower tear break-up time with BAK containing eye drops^48^,^49^ **Ocular surface alterations** – Conjunctival epithelial changes^50^ – Corneal alteration	**Improved tear film stability** – Improvement of tear break-up time with both vehicle and Cyclokat after three months of treatment **Improved ocular surface** – Improvement of corneal staining with both vehicle and Cyclokat after six months of treatment^51^	**Improved tear film stability** – Improvement of tear break-up time with Cationorm and Cyclokat **Improved ocular surface** – Improvement of corneal staining with Cationorm and Cyclokat

BAK, benzalkonium chloride; CKC, cetalkonium chloride.

The CKC cationic o/w nanoemulsion was used as a vehicle for lipophilic drugs such as ciclosporin or latanoprost. Nonclinical studies performed in the rabbit or in the rat demonstrated that these ophthalmic drug products were safe and well tolerated following single and repeated applications, with neither inflammatory cell infiltration nor apoptosis.^47^,^52^–^55^ This good safety profile was confirmed by clinical trials for both drug products, and by the commercialization (since 2008) of the CKC cationic o/w nanoemulsion vehicle (Cationorm) as an improved artificial tear substitute for the relief of mild to moderate dry eye symptoms.^17^,^56^–^59^

## Protective properties of the cationic oil-in-water nanoemulsion vehicle

The increased residence time and better spreading properties of the cationic o/w nanoemulsion designed to improve the ocular bioavailability of lipophilic drugs was accompanied by unexpected beneficial effects for the ocular surface.

**Figure 5:**
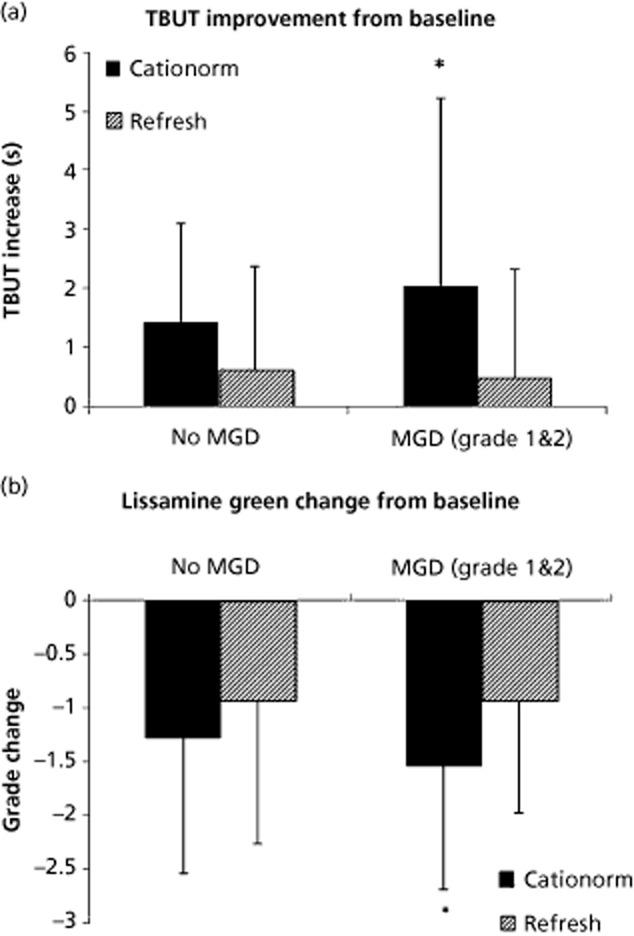
Tear film break-up time and lissamine green staining in dry eye patients with or without meibomian gland dysfunction (MGD) treated with Cationorm or Refresh. (a) Tear break-up time (TBUT) improvement over time, and (b) lissamine green change from baseline following Cationorm or Refresh treatment. *P < 0.05 (generalized estimating equation models, analysis of variance).

Applications of the cationic o/w nanoemulsion help restore the integrity of the lacrimal film through the concomitant action of the oil and the slightly hypoosmolar aqueous phase. The oil phase of the cationic o/w nanoemulsion by mixing with the tear film lipid layer contributes to its stability, thus reducing the evaporation of water from the aqueous phase. This is of particular interest for meibomian gland dysfunction (MGD) patients with short tear film break-up times (TFBUTs) due to the lipid deficiency of their tears. The cationic o/w nanoemulsion vehicle Cationorm was able to improve keratitis (corneal fluorescein staining), TFBUT, and significantly reduced the symptoms of dry eye disease (DED) (Figure 5).^60^ The TFBUT was significantly greater with Cationorm than with Refresh in eyes with MGD. Cationorm was even better in MGD condition than in non-MGD, suggesting a positive correlation, while Refresh showed no better efficacy in eyes with MGD.

Thus, by mechanically stabilizing the tear film the cationic o/w nanoemulsion confirmed its benefits for the relief of mild to moderate dry eye.^17^ Hyperosmolarity of the tear is known to be pro-inflammatory, thus the hypoosmolarity of the aqueous phase may contribute to the management of DED signs and symptoms by transiently (upon instillation) normalizing tear osmolarity post instillation.^61^,^62^

**Figure 6:**
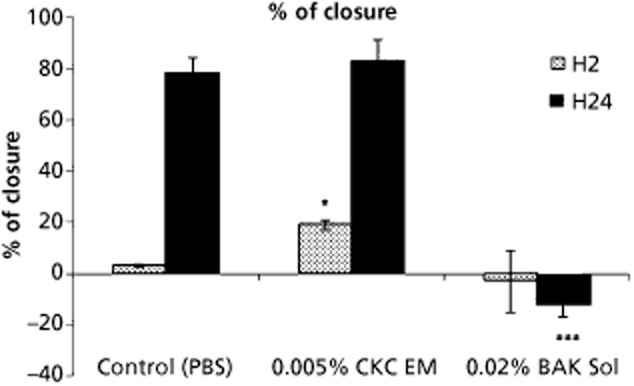
In-vitro scraping assay (human corneal epithelial cells) measuring the pace of the healing process following a 30-min treatment with 1/10 dilutions of 0.005% cetalkonium chloride cationic oil-in-water nanoemulsion (0.005% CKC EM) or 0.02% benzalkonium chloride solution (0.02% BAK Sol). *P < 0.05 against phosphate buffered saline (PBS); ***P < 0.01 against PBS (two-way analysis of variance followed by Fisher adjustment). H2, two hours; H24, 24 hours.

Adapted from Liang *et al*.^55^.

**Figure 7:**
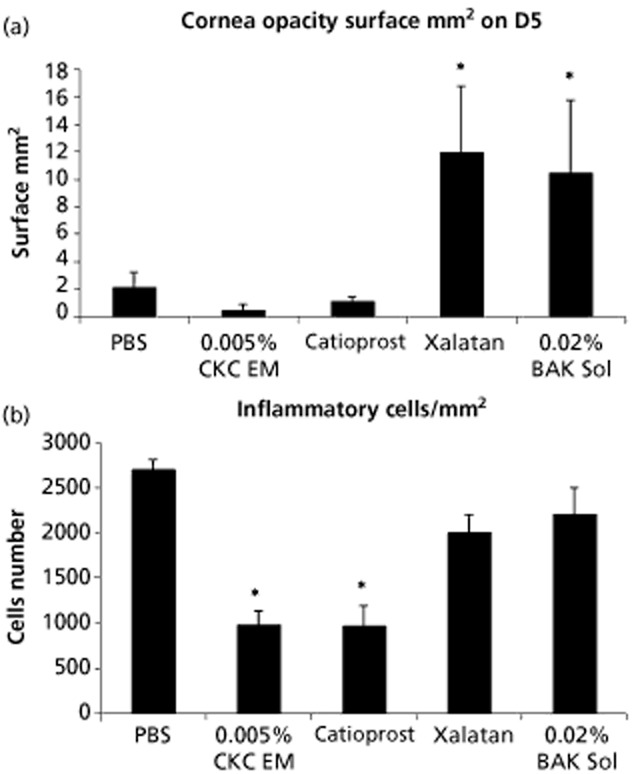
(a) Scar size and (b) inflammatory cell count in the rat cornea at day 5 (D5), after corneal scraping and five days treatment with cationic oil-in-water nanoemulsion. 0.02% Benzalkonium chloride solution (0.02% BAK Sol); 0.005% cetalkonium chloride cationic oil-in-water nanoemulsion (0.005% CKC EM). *P < 0.05 against phosphate buffered saline (PBS) (two-way analysis of variance followed by Fisher adjustment).

Adapted from Liang *et al*.^55^.

More surprising were the beneficial effects of the cationic o/w nanoemulsion on the wound healing process. Repeated instillations of the cationic o/w nanoemulsion Cationorm were demonstrated to help the wounded corneal epithelium recover faster than following treatments with conventional artificial tears in a rabbit model of corneal abrasion.^17^ Both in-vitro and in-vivo data demonstrated that the CKC cationic o/w nanoemulsion promoted wound healing.^55^ On scraped human corneal epithelial (HCE) cells a 30 min application of the cationic o/w nanoemulsion was able to increase the pace of healing, as measured by the reduction of the size of the scraped area (Figure 6). These in-vitro data were confirmed in-vivo in a rat model of corneal scraping. Following corneal mechanical abrasion, a twice daily treatment with the CKC cationic o/w nanoemulsion allowed for a complete and almost scar-free re-epithelization of the cornea (Figure 7a).^55^ By opposition, treatment with a 0.02% BAK aqueous solution resulted in the formation of an opaque scar underneath the healed epithelium. These data suggested that the CKC cationic o/w nanoemulsion was able to promote a safe healing process, without the formation of opaque scar tissue within the cornea, as if the CKC cationic o/w nanoemulsion was able to manage the inflammation that resulted from the initial mechanical corneal abrasion. The number of inflammatory cells was then determined on fixed and haematoxylin-eosin stained rat corneas (Figure 7b). Corneas from the CKC cationic o/w nanoemulsion-treated group presented a reduced number of inflammatory cells, while in the other groups (phosphate buffered saline- or 0.02% BAK aqueous solution-treated groups) the inflammatory cell count remained elevated. This clearly suggested that the CKC cationic o/w nanoemulsion may have harboured anti-inflammatory properties.^55^ The same results were obtained with Cationorm, which contains 0.002% CKC as the cationic agent, and was confirmed in the rabbit following repeated instillations of a 0.05% ciclosporin cationic o/w nanoemulsion.^52^,^63^ The mechanism underlying these observations is currently under evaluation.^64^

## Conclusions

Cationic o/w nanoemulsions represent a new development strategy to improve ocular drug delivery of lipophilic compounds. The main issue in the development of these products was the choice of the cationic agent. CKC was found to be the best cationic agent to produce stable unpreserved cationic o/w nanoemulsions with unexpected beneficial biological activity for the ocular surface. The use of CKC over BAK as cationic agent appears obvious (Table 3) when comparing the physicochemical properties of these compounds. In the continuous effort to improve ocular drug delivery a CKC cationic o/w nanoemulsion was developed to improve the precorneal residence time and the spreading properties on negatively charged ocular surface cells of the nanoemulsions. This better spreading and improved residence time of the CKC cationic o/w nanoemulsion translated into a twofold increase in ciclosporin ocular bioavailability over anionic ciclosporin nanoemulsions. This new type of vehicle was demonstrated to be perfectly safe and well tolerated by the ocular surface. While BAK in conventional aqueous eye drops has a preservative role, thanks to its detergent action, CKC in the cationic o/w nanoemulsion exhibits neither detergent effect nor preservative role. Consequently, CKC cationic o/w nanoemulsion does not exhibit any of the observed ocular side effects related to BAK in aqueous eye drops (tear film instability, ocular surface damage, mucus removal). In addition to the improved bioavailability of the loaded drug, these CKC cationic o/w nanoemulsions have ocular surface protective properties through the restoration of a healthy tear film and by favouring the corneal wound healing process through the promotion of re-epithelization and inflammation management.

## Declarations

### Conflict of interest

The authors are employees of Novagali Pharma SAS.

### Funding

This review received no specific grant from any funding agency in the public, commercial, or not-for-profit sectors.
